# Fréquence et titrage des hémolysines anti-A et anti-B chez les mères d’enfants ictériques à Yaoundé, Cameroun

**DOI:** 10.11604/pamj.2020.35.13.14770

**Published:** 2020-01-15

**Authors:** David Sock Sock, Severin Donald Kamdem, Angeline Boula, Palmer Masumbe Netongo

**Affiliations:** 1 Ecole des Sciences de la Santé, Université Catholique d’Afrique Centrale, Yaoundé, Cameroun; 2 Molecular Diagnostic Research Group, the Biotechnology Centre, University of Yaoundé I, Yaoundé, Cameroon; 3 Centre Mère et Enfant de la Fondation Chantal Biya, Yaoundé Cameroun; 4 Département de Biochimie, Université de Yaoundé, Yaoundé, Cameroun

**Keywords:** Hémolysines anti-A et anti-B, ictères néonataux, incompatibilité fœto-maternelle, système ABO, Anti-A and anti-B hemolysins, neonatal jaundice, feto-maternal incompatibility, ABO system

## Abstract

**Introduction:**

L’allo-immunisation du système ABO est impliquée dans les ictères néonataux avec une prévalence globale considérable. Le rôle de l’incompatibilité dans le système ABO est relativement peu connu. L’objectif de cette étude était d’investiguer les ictères néonataux attribuable aux incompatibilités fœto-maternelles dans le système ABO et de déterminer le lien entre le titre d’hémolysines retrouvé chez la mère et le degré d’ictère observé chez l’enfant.

**Méthodes:**

Nous avons mené une étude transversale de juin à novembre 2015 et la population d’étude était exclusivement constituée des mamans de groupe sanguin « O » ayant des enfants de groupe sanguin différents reçus en service de néonatalogie des centres hospitalier de référence dans la ville de Yaoundé. Les analyses statistiques ont été réalisées à l’aide du logiciel GraphPadPrism 6 à un intervalle de confiance de 95%.

**Résultats:**

La fréquence d’hémolysines retrouvées dans cette étude était de 20,58% (7/34) et l’hémolysine anti-A était le plus fréquent avec 85,7% (6/7). Les nouveau-nés de groupe sanguin B ont présenté une plus grande concentration en bilirubine comparer à ceux du groupe AB (p = 0,01). La multiparité n’était pas associée à la présence d’hémolysine (p = 0,8) tout comme le groupe sanguin de l’enfant n’a été associé à la survenue des hémolysines chez la mère (p = 0,5).

**Conclusion:**

Les ictères néonataux précoces ou prolongés sont également causés par des hémolysines anti-A et anti-B dérivées de l’allo-immunisation ABO. Une étude sur un échantillonnage plus grand est recommandée pour une meilleure évaluation.

## Abstract (en)

**Introduction:**

The alloimmunization of the ABO blood group system is involved in neonatal jaundice with a considerable overall prevalence. The role of ABO incompatibility is relatively little known. The purpose of this study was to investigate neonatal jaundice due to feto-maternal ABO incompatibilities and to determine the link between the hemolysins value in the mother and the degree of jaundice observed in the infant.

**Methods:**

We conducted a cross-sectional study from June to November 2015. The study population was exclusively composed of moms who were blood type O with children who were a different blood type hospitalized in the Department of Neonatology at the Reference Hospital in the city of Yaoundé. Statistical analyses were performed using the GraphPadPrism 6 software with a confidence interval of 95%.

**Results:**

Hemolysins frequency was of 20.58% (7/34) and anti-A hemolysin was the most common type (85.7%; 6/7). The new-born who had blood type B had a greater concentration of bilirubin levels compared to those of the AB group (p = 0.01). Multiparity was not associated with the presence of hemolysin (p = 0.8) as well as blood type of the infant was not associated with the occurrence of the hemolysins in the mother (p = 0.5).

**Conclusion:**

Early neonatal jaundice or protracted neonatal jaundice are also caused by hemolysins anti-A and anti-B derived from the allo-ABO immunization. A study on a larger sample is recommended for better assessment.

## Introduction

Les incompatibilités fœto-maternelles érythrocytaires (IFME) sont définies par la fixation d’allo anticorps maternels sur les globules rouges du fœtus, anticorps transmis pendant la grossesse et qui ont pour cible les antigènes de groupes sanguins du fœtus, d’origine paternelle [1]. Les complexes immuns ainsi formés provoquent une immuno-hémolyse tissulaire. Chez le nouveau-né, le syndrome hémolytique s’exprime de deux façons: une anémie qui peut débuter in utero et se prolonge pendant plusieurs semaines après la naissance, et une hyperbilirubinémie (ictère) précoce et rapidement croissant après la naissance [2]. Les ictères néonataux quant à eux sont d’observation courante (65 à 70%) [3] dans les services de maternité et de néonatologie. Ils sont associés à la morbidité et à la mortalité infantile. Ils représentent la cause la plus commune de réadmission des nouveau-nés à l’hôpital [4]. Ces ictères néonataux peuvent entraîner l’accumulation de la bilirubine dans les ganglions fondamentaux et dans le cerveau et causer un ictère nucléaire. Si les victimes de cette phase aigüe parviennent à survivre, à la longue ils peuvent développer une encéphalopathie chronique. Cette condition est marquée par une surdité et un retard mental [5].

Le diagnostic des ictères revient en pratique à celui d’exclusion. La prise en charge reste une importante activité des pédiatres de maternité et des professionnels de la santé dans les services de néonatalogie [6-9]. Les ictères hémolytiques par incompatibilité fœto-maternelle connaissent deux étiologies dominantes: premièrement, l’immunisation de la mère rhésus négatif contre l’antigène D. Celle-ci est la plus connue et la mieux étudiée. Elle devient rare de nos jours grâce à une prophylaxie systématique. Deuxièmement l’immunisation dans le système ABO (cas d’une mère O portant un fœtus de groupe A, B ou AB). Cette dernière, non seulement elle représente la plus grande cause des maladies hémolytiques néonatales actuellement, mais est relativement peu connue [2]. Contrairement aux anticorps anti-A et anti-B naturels, les anticorps immuns sont fortement hémolysants par leur capacité à déclencher la cascade complète du complément. On parle ainsi d’hémolysines. Ils sont en outre caractérisés par un maximum d’activité à 37°C et sont de nature IgG. Ces derniers sont capables de traverser la barrière fœto-placentaire et entraîner la maladie hémolytique du nouveau-né. Ils sont difficilement absorbables par les antigènes A et B solubles [10, 11].

Par ailleurs, il a été suggéré que la plus grande fréquence des maladies hémolytiques du nouveau-né (MHNN) chez les africains est due à la très haute activité des hémolysines anti-A et anti-B des individus de groupe sanguin O [12, 13]. Les quelques travaux qui lui sont consacrés en Afrique Noire montrent qu’elle est très fréquente avec des conséquences parfois sévères [14] chez environ 5% de tous les nouveau-nés [15]. Toutefois, une étude effectuée au Maroc a montré que les étiologies des ictères néonataux sont dominées par l’incompatibilité ABO avec un pourcentage de 27,5% [16]. En plus, 62,2% des 1686 enfants ont développé l’ictère dans une étude menée à Lagos au Nigeria. Parmi ces ictères, 30% étaient associés à l’allo immunisation du système ABO [17]. Au Cameroun, les résultats d’une étude sur la recherche et le titrage des hémolysines anti-A et anti-B chez les femmes en période du postpartum immédiat au Centre Hospitalier et Universitaire de Yaoundé (CHUY) ont révélé que la fréquence de l’incompatibilité fœto-maternelle ABO était de 22,7% et celle des hémolysines était de 26,8%. Les hémolysines anti-A, anti-B et anti-AB ont été retrouvées respectivement dans 15,0%, 18,7% et 6,9% des cas [18]. Au vu de ces résultats, il s’était avéré intéressant de rechercher la fréquence de ces hémolysines chez les femmes dont les nouveau-nés à terme étaient victime d’ictères précoces et prolongés (ictère persistant jusqu’à 11 jours de vie) en rapport avec les incompatibilités fœto-maternelles et établir un lien entre le taux d’hémolysine chez la maman et le degré d’ictère chez l’enfant. Ceci dans le but d’augmenter la capacité de diagnostic et de prise en charge des ictères hémolytiques du nouveau-né liés aux incompatibilités dans le système ABO.

## Méthodes

**Type et lieu de l’étude:** nous avons mené une étude transversale dans les services de néonatalogie de quatre hôpitaux de références de la ville de Yaoundé au Cameroun à savoir: le Centre Mère et Enfant de la Fondation Chantal Biya (CME, FCB); l’Hôpital Gynéco-Obstétrique et Pédiatrique de Yaoundé (HGOPY); l’Hôpital Général de Yaoundé (HGY), et enfin le Centre Hospitalier d’Essos (CHE).

**Population de l’étude:** la population d’étude était constituée de toute femme de groupe sanguin « O » au service de néonatalogie ayant un ou plusieurs nouveau(x)-né(s) à terme et de groupe sanguin diffèrent « A, B, ou AB » présentant un ictère précoce (apparition dans les 24 heures de vie) ou prolongé (persistant jusqu’au 11^ème^ jour de vie).

**Taille et duré de l’étude:** nous avons administré un consentement éclairé chez 180 mamans dont leurs enfants étaient atteints d’ictère, et seul 34 mères et leurs 34 enfants ont été retenus pour cette étude qui a duré 06 mois (juin à novembre 2015).

**Collecte de sang:** le sang veineux a été recueilli chez tous les participants (mères et enfants). Chez les enfants, 2ml de sang total a été collecte dans des tubes d’acide éthylènediaminetétraacétique (EDTA) pour le dosage de la bilirubine (directe et totale), la détermination du groupe sanguin, et la réalisation du Coombs direct. Chez les mamans, 2ml de sang total a été collecte dans des tubes EDTA pour la confirmation du groupe sanguin et la recherche des hémolysines. Le transport des échantillons des différents hôpitaux pour le lieu d’analyse (Laboratoire Centre Mère et Enfant de la Fondation Chantal Biya) s’effectuait à l’aide des portoirs à tubes préalablement emballé avec un papier absorbant et incorporé dans une boite hermétiquement fermée et à une température ambiante (+20°C à +24°C) (OMS, 2008). Le tube de l’enfant était isolé de la lumière à l’aide d’un papier aluminium et conservé dans une boite noire. En cas d’examens différés, le plasma réservé à la recherche des hémolysines était conservé à -20°C pendant un maximum de deux semaines (Louati, 2008). Par ailleurs, celui destiné au dosage de la bilirubine était conservé à l’abri de la lumière à -20°C pendant deux semaines selon (le kit CYPRESS diagnostic). La valeur du taux de la bilirubine indirecte était déterminée par la différence entre le taux de bilirubine totale et le taux de bilirubine directe.

**Considération éthique:** une clairance éthique a été obtenue auprès du Comité National d’Ethique en Recherches en Santé Humaine (clairance No 199/CIERSH/DM/2015) suivit des autorisations de recherches auprès de tous les responsables d’hôpitaux. Les consentements éclairés ont été obtenu auprès des parents avant inclusion (parents et enfants) dans l’étude.

**Analyses statistiques:** les données collectées ont premièrement été introduit dans un fichier Excel. Ensuite, les graphes et analyses statistiques ont réalisé à l’aide du logiciel GraphPadPrism 6. Les moyennes, écart types, fréquences et pourcentages ont été utilisé pour présenter les données. Le *Student t-test* a été utilisé pour comparer des variables entre 2 groupes. Toutes les analyses statistiques ont été réalisées à un intervalle de confiance de 95% et toute p-values < 0,05 était considérée significative.

## Résultats

**Figure 1:**
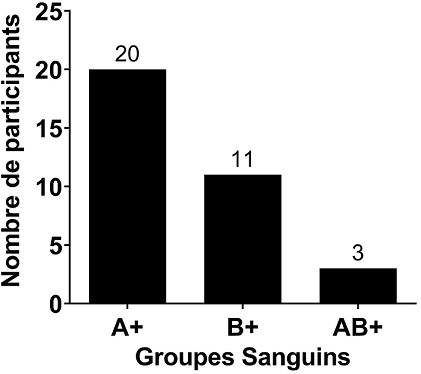
Groupes sanguin des nouveau-nés

**Figure 2:**
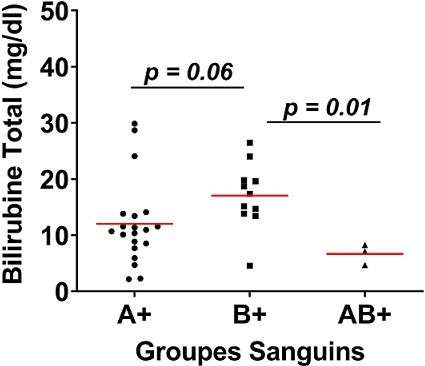
Concentration bilirubine total par groupe sanguin

**Figure 3:**
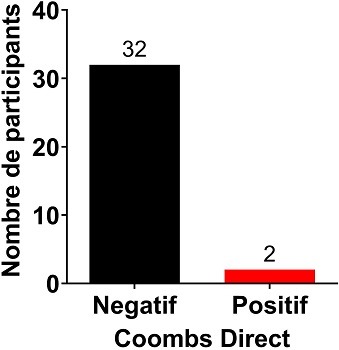
Faible taux de positivité de Coombs direct chez les nouveau-nés

Au total, 180 prélèvements ont été réalisés chez les enfants atteints d’ictère et leurs mamans. Après analyse au laboratoire, seuls 34 paires de prélèvements mamans / enfants ont été retenus. Des 34 mères incluses, l’âge moyen était de 24,68 ans. La maman la moins âgée avait 17 ans tandis que la plus âgée avait 37 ans. La majorité des mères avait un âge compris dans l’intervalle 24,68 ± 4,77 ans avec un pourcentage de 61,76% (21/34). Les primipares (21/34) étaient les plus représentées avec une proportion de 61,76% (Tableau 1). L’âge des nouveaux nés variait de 01 à 10 jours avec une moyenne de 4,32 ± 2.19 jours. Avec une proportion de 58,82% (20/34), le sexe féminin était le plus représenté devant le sexe masculin 41,18% (14/34) (Tableau 2). Le groupe sanguin A était le plus représenté avec un pourcentage de 55,82% (20/34) devant le groupe B 32,35% (11/34) et le groupe AB 8,82% (3/ 34) (Figure 1). Le taux de bilirubine indirecte était significativement plus élevé chez les nouveau-nés de groupe sanguin B compare à ceux du groupe sanguin AB (p = 0.01). Bien que le taux de bilirubine fût plus élevé dans le groupe B comparer au groupe sanguin A, la différence n’était pas significative; les nouveau-nés de groupe sanguin B présentaient un taux de bilirubine plus élevé que ce du groupe sanguin A et AB (Figure 2). Sur 34 prélèvements chez des enfants, 2 Coombs directes se sont avérés positif soit 50% pour le groupe A et 50% pour le groupe B (Figure 3).

**Tableau 1:** Données sociodémographiques des mères

Ages (Ans)			Nombre de grossesses		Statut Matrimonial		Durée grossesse (semaines)		
Min	Moy	Max	1	[2 et plus]	Cel	Mar	36	37	38
17	24,67	37	21	13	25	11	28	04	02
**Pourcentages (%)**	61,76	38,24	73,50	26,50	82,30	11,80	5,90

Min: Minimum; Moy: moyenne; Max: Maximum; Cel: Celibataire; Mar: Mariée

**Tableau 2:** Données sociodémographiques des enfants

Ages (Jours)			Sexes (%)		Groupes sanguins		
Min	Moyenne	Max	Masculin	Féminin	A+	B+	AB+
01	4,32	10	41,18	58,82	20	11	03

### Résultats des hémolysines par groupe sanguin

**Figure 4:**
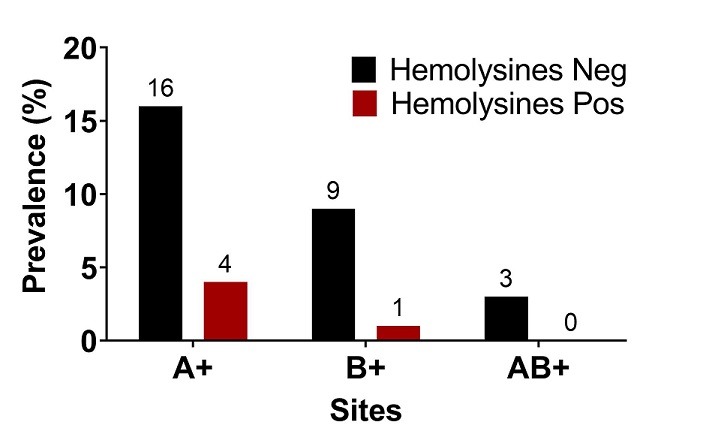
Titre d’hémolysine par groupe sanguins

**Figure 5:**
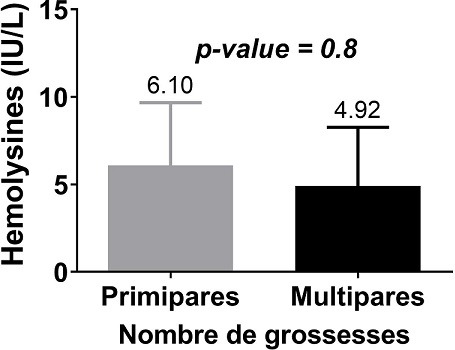
Titre d’hémolysine par nombre de grossesse

**Figure 6:**
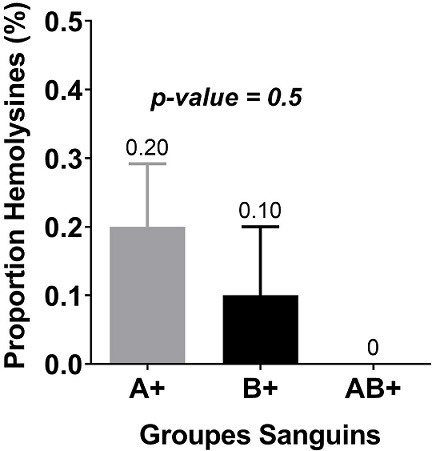
Relation entre la présence d’hémolysines et le groupe sanguin

Les hémolysines étaient plus représentées chez les mamans ayant les enfants de groupe A avec un pourcentage de 8,82% (3/34) devant celle de groupe B dont le pourcentage s’élevait à 2,94 (1/34) (Figure 4). Chez les primipares qui était les plus représenté avec 21 mères, 11,76% possédaient des hémolysines anti-A pour des titres variant de 32 à 64µg/mL. Cependant, aucune primipare ne possédait des hémolysines anti-B. Chez les multipares représentés par 13 mères, 08,82% possédaient uniquement des hémolysines anti-B pour des titres variant de 32 à 64µg/mL. Aucune multipare ne possédait des hémolysines anti-A et le nombre de grossesse n’avait pas d’influence (*p-value* = 0,8) sur la présence des hémolysines anti-A et anti-B (Figure 5). Enfin, le groupe sanguin des enfants n’était pas associé à la survenue des hémolysines chez les mamans (*p-value* = 0,5). Les hémolysines anti-A et anti-B sont représentées semblablement chez les mères primipares et multipares (Figure 6).

## Discussion

Cette recherche visait à déterminer et à quantifier les hémolysines chez les mères essentiellement de groupe sanguin « O » dont les enfants de groupe sanguin A, B ou AB, présentaient un ictère précoce ou prolongé. Nous avons retenue dans cette étude 34 couples (mères et enfants). Cette taille est inférieure à celle (246 femmes) utilisée par Fopa *et al.* en 2014 [18] au Centre Hospitalier et Universitaire de Yaoundé sur la recherche et le titrage des hémolysines anti-A et anti-B chez les femmes en postpartum immédiat ainsi que les facteurs de risque associés. Ceci s’explique par le fait que ces auteurs recherchaient les hémolysines chez toutes les femmes en postpartum immédiat et en situation d’incompatibilité sanguine dans le système ABO ne tenant pas compte de l’ictère de l’enfant. Par conséquent, la présente étude incluait uniquement les femmes qui avaient des enfants ictériques.

De plus, les 34 enfants ictériques retenus pendant une période de 06 mois montre l’extrême rareté des maladies hémolytiques du nouveau-né à Yaoundé au Cameroun. Cette rareté est confirmée par une étude menée à Sarajevo en 2013, qui a révélé que dans les incompatibilités ABO, 15% de grossesses des mères de groupe sanguin O donnent naissance à des enfants de groupe A ou B. Selon cette étude, la fréquence des maladies hémolytiques du nouveau-né liée au système ABO est rare et a lieu dans 1/150 grossesses [19]. En outre, dans cette étude, la disparité ABO représente 27,22% de la totalité des échantillons prélevés. Ceci est semblable aux rapports de Badiee [20] et Sanpavat [21] qui ont rapporté les incompatibilités ABO dans les proportions respectives de 22,0% et 21,3% de cas et aussi par les travaux de Izetbegovic en 2013 [1] pour qui 20% des nouveau-nés sont de système ABO incompatible avec la mère. Seul parmi ceux-ci 1 enfant sur 60 aura des manifestations d’hémolyse justifiant une intervention thérapeutique.

Les nouveau-nés les plus représentés dans cette étude sont ceux de 02, 03 et 04 jours de vie avec des pourcentages d’apparition respectifs de 17,65%, 20,59%, 23,53%. Ceci pourrait s’expliqué par la non reconnaissance de l’ictère et/ou par la négligence de celui-ci dans ces débuts. Ceux-ci étaient réadmis dans les services de néonatalogie lorsque la gravité était prononcée. Ceci concorde également avec les travaux de Poissonnier [15] qui atteste que le diagnostic clinique est typiquement évoqué devant l’ictère précoce (avant 48 heures de vie). Il s’accentue jusqu’au 3^ème^ ou 4^ème^ jour, sans pâleur notable. Selon cet auteur, certaines formes d’ictères évoluent sous la forme d’ictère très précoce et intense mais rapidement résolutif. Inversement, l’hyperbilirubinémie peut s’accentuer tout au long de la première semaine [15].

Dans la présente étude, aucune transfusion sanguine n’a été enregistrée, or celle-ci peut également être à l’origine des hémolysines chez les mères d’enfants. Car les études de Jaisy Mathai [22] révèlent que les hémolysines causent les hémolyses chez le receveur et sont connues par leur capacité à traverser la barrière placentaire et par conséquent, pourraient être à l’origine de la maladie hémolytique du nouveau-né [23]. Tous les enfants dans cette étude présentaient un ictère. Ainsi, les moyennes des taux de bilirubines directe, totale et indirectes étaient respectivement de 1,4718 ± 1,95 mg/dl; 13,17 ± 7,29 mg/dl et de 11,7018 ± 6,8308 mg/dl. Ceci concorde avec les résultats de Rambaud qui attestent que l’ictère apparaît pour des valeurs de bilirubinémie supérieures à l’intervalle (03 - 04,7) mg/dl [24].

Sur 180 couples (mère-enfant) de prélèvement, 49 (27,22%) de ces derniers se sont avérés incompatibles dans le système ABO (maman de groupe O et enfant de groupe A, B ou AB). Ainsi 34 (18,88%) couples ont été retenus. Parmi ceux-ci, 05 (14.70%) cas d’hémolysines ont été détectés avec 02 tests de Coombs positifs. Ce qui concorde avec les travaux qui ont montré que les ictères ne sont pas seulement causés par les hémolysines [25-27], mais que les infections bactériennes constituaient sans doute la cause la plus commune des ictères néonataux [28], suivie des atrésies du canal biliaire [29].

Les hémolysines anti-A étaient les plus représentés soit 11,76% par rapport aux anti-B avec 2,94%. Ces résultats concordent avec ceux de Fopa *et al.* en 2014 au CHUY [18] qui attestent aussi que les titres les plus élevés étaient ceux d’hémolysines anti-A. Par ailleurs, le titre le plus élevé concernait l’hémolysine anti-A avec une valeur de 64 µg/mL. D’après Poissonnier, après dénaturation des IgM le titre sérique des hémolysines anti-A ou anti-B en milieu physiologique est compris entre 32 et 128, il dépasse 1 024 dans les formes les plus sévères [15].

Dans la présente étude, le groupe sanguin de l’enfant n’a aucun impact sur la survenue des hémolysines anti-A et anti-B avec une *p-value* = 0,5. Ce résultat infirme celui de Fopa *et al.* en 2014 [18] pour qui l’incompatibilité fœto-maternelle ABO représente un facteur de risque. De même, aucun lien entre le nombre de grossesses et la présence des hémolysines anti-A et anti-B montrés n’a été trouvé (*p-value* = 0,8). Ceci a également été trouvé par Poissonnier [15], et Sebija Izetbegovic [1]. Selon ces auteurs, l’immunisation anti-A ou anti-B de la mère, qui est dans 95% des cas de groupe O, est liée à l’existence de substances ubiquitaires proches des antigènes A et B. C’est pourquoi la maladie hémolytique par IFM ABO affecte un premier né dans 40% des cas. Elle traduit l’hémolyse des hématies du nouveau-né présentant l’antigène homologue [1]. Par ailleurs, Jaisy Mathai *et al.* dans une étude en Inde en 2002 [22] ont fait le teste des hémolysines pour la caractérisation de l’immunité des anticorps du système ABO. Ils ont trouvé que le plus haut niveau d’hémolysines dans ces populations était corrélé aux piqures des moustiques et aux parasites intestinaux.

Les hémolysines retrouvées chez les femmes primipares (primigestes) peuvent être dues à une hétéro-immunisation, notamment par produits médicamenteux, en particulier vaccins, sérums tels l’anatoxine diphtérique ou tétanique [22]. En effet, ce phénomène peut également survenir, du fait de la répétions des HFM. Le rang de grossesse ne semble pas influer sur la gravité de l’expression clinique des IFME ABO [2]. Dans notre population étudiée, l’analyse de la variance nous a permis de conclure qu’il n’ya pas de lien statistiquement significatif entre les hémolysines du sang maternel et le degré d’ictère chez l’enfant. Ceci s’explique par la relativité de l’immunogénécité des antigènes des globules rouges du fœtus et de l’antigenécité des hémolysines synthétisées par la maman comme l’ont démontré Tissier *et al.* en 1996. Nous convenons ainsi avec Cortey pour qui, la faible antigénicité ABO des érythrocytes fœtaux et l’existence de ces mêmes sites antigéniques dans d’autres systèmes cellulaires réduisent la gravité de ce type d’allo-immunisation [2].

## Conclusion

Au terme de cette étude dont l’objectif général était de mettre en évidence le rôle des hémolysines anti-A et anti-B dans les incompatibilités fœto-maternelles en relation avec les ictères néonataux dans le contexte camerounais et précisément à Yaoundé. Nous avons notez une grande proportion d’hyper bilirubinémie dans notre population cibles, demandant beaucoup plus de vigilance de la part des personnels soignant. De même, les incompatibilités dans le système ABO entre la mère de groupe sanguin O et l’enfant de groupe sanguin A, B et AB ne sont pas à négliger. Cependant, nos résultats suggèrent que le groupe sanguin de l’enfant n’a aucun effet sur la présence/fréquence et les titres des hémolysines anti-A et anti-B chez les mamans. De plus, le groupe sanguin des enfants n’a pas été associé au degré d’ictères néonataux précoces ou prolongés chez ces derniers.

### Etat des connaissances actuelles sur le sujet

- La connaissance du groupe sanguin du couple mère-enfant, le test d’hémolysine chez la mère et le test de Coombs directe chez l’enfant le premier jour de vie sont importants pour la prévention et la prise en charge des ictères liés aux incompatibilités fœto-maternels dans le système ABO.

### Contribution de notre étude à la connaissance

- Cette étude a un intérêt en santé publique, spécifiquement dans le domaine des incompatibilités fœto-maternels dans le système ABO; ce travail devrait pouvoir aider à comprendre les risques de survenue des mariages hétéro groupés dont la femme est de groupe sanguin « O »;
- Ainsi ce travail devrait donc pouvoir aider les couples et le personnel soignant à s’attendre à un ictère si à la naissance le groupe sanguin de l’enfant et différent du groupe sanguin « O » de la mère;
- En fin ce travail devrait renseigner le personnel soignant à instaurer la systématisation des groupages sanguins des mamans et leur(s) nouveau(x)-né(s), et de systématiser le teste de Coombs direct de l’enfant si le groupe sanguin de l’enfant est différent du groupe sanguin « O » de la mère.

## Remerciements

Les auteurs remercient le groupe de recherche « Molecular Diagnostic Research Group » pour la contribution à l’élaboration de ce travail, ainsi que Dr NGO SOCK Emilienne Tudor pour son appui intellectuel et financier.
